# On the Microstructure, Strength, Fracture, and Tribological Properties of Iron-Based MMCs with Addition of Mixed Carbide Nanoparticulates

**DOI:** 10.3390/ma13132892

**Published:** 2020-06-27

**Authors:** Grzegorz Królczyk, Eugene Feldshtein, Larisa Dyachkova, Mariusz Michalski, Tomasz Baranowski, Roman Chudy

**Affiliations:** 1 Faculty of Mechanical Engineering, Opole University of Technology, Mikolajczyka Str. 5, 45-271 Opole, Poland; r.chudy@po.edu.pl; 2 Faculty of Mechanical Engineering, University of Zielona Góra, Prof. Z. Szafrana 4, 65-516 Zielona Góra, Poland; e.feldsztein@ibem.uz.zgora.pl (E.F.); M.Michalski@ibem.uz.zgora.pl (M.M.); T.Baranowski@ibem.uz.zgora.pl (T.B.); 3 Powder Metallurgy Institute, Belarusian National Academy of Sciences, ul. Platonova 41, 220005 Minsk, Belarus; dyachkova@tut.by

**Keywords:** metal-matrix composites (MMCs), ceramic nanoparticulates, mechanical properties, fractures, tribological behavior, wear

## Abstract

In this paper, the features of the strength, fractures, and tribological behavior of metal-matrix composites based on the FeGr1 material are discussed. To improve the material properties, a mixture of SiC, Al_2_O_3_ and C nanoparticulates have been added to an iron-based matrix. The simplex lattice design method and hardness, compression, and bending tests were used to determine the mechanical properties. Scanning electron microscopy was applied for fracture features analysis. Different fracture types, mainly trans-crystalline quasi-brittle and brittle fracture or inter-granular fracture and microcracks were registered for the composites tested. Depending on the type and amount of ceramic additives, significant changes in strength, as well as in the fracture features of the metal-matrix composites (MMCs), were observed. Based on tribological tests, changes in the momentary coefficients of friction, temperature of the friction surface, and wear rate of the composites with nanoparticulates were described. An analysis of the worn surface morphology revealed changes in the wear process depending on the MMC composition. It was shown that the use of hybrid mixed additives based on hard ceramic nanoparticulates improved both strength and tribological properties of composites.

## 1. Introduction

Composites are important in the development of many industries, particularly transport, engineering, or military technology. As a base material, polymers, metals, and various ceramics are used. Aluminum, titanium, copper, and iron are widely used as the matrix of metal-based composites. According to Chaudhari and Singh [1], different oxides, particularly alumina Al_2_O_3_, zirconia ZrO_2_, silica SiO_2_, tungsten oxide WO_3_, chromium oxide Cr_2_O_3_, titanium oxide TiO_2_, yttria Y_2_O_3_, and many others, can be used as hardening additives. On the other hand, different carbides, particularly SiC, WC, B_4_C, and TiC, have been in widespread use for a long time. Reinforcing additions may be used as separate particulates, as particulate complexes, continuous fibers, short fibers, or whiskers.

Composites based on aluminum are in widespread use in mechanical engineering. It was found by Bharath et al. [2] that the introduction of Al_2_O_3_ particulates in Al-based materials provided a good distribution of particulates in the materials studied. Increasing the content of alumina particulates ensured increases in the hardness and tensile strength of metal-matrix composites (MMCs). The Al_2_O_3_ particulates located in the Al matrix composites significantly increased their wear resistance. Pramanik [3] revealed that these particulates prevented the abrasion and limited surface deformations of materials. The occurrence of alumina particulates and their sizes are the most significant factors when affecting the fatigue strength of Al-based composites, as it was shown by Pramanik et al. [4]. Larger particulates hindered the growth of cracks or changed their movement. Selvakumar et al. [5] considered Al–Cu composites with SiC additives. It was shown that with an increase in the content of silicone carbides, the microhardness and compression strength increased, but the thermal conductivity decreased. Wear resistance increased with increasing SiC content. According to Pramod et al. [6], composites based on Al7075 alloy with Al_2_O_3_ additives showed an increase in the ultimate load and friction path compared to the base aluminum alloy. Nonlinear dependences of the density, friction path, and wear on the weight content of alumina were revealed. Studies of Al-based MMCs with Al_2_O_3_ particulates described by Kumar et al. [7] revealed the presence of silicon in the needle formed at the grain boundaries of the matrix. It was shown that with a decrease in the size of alumina, the tensile strength and hardness of composites increased, but at the same time, the relative elongation decreased. The presence of alumina affected the wear resistance of composites studied, and while reducing the Al_2_O_3_ size, the resistance to wear increased. It was shown by Kumaraswamy et al. in [8] that the introduction of alumina, silicon carbide, and boron carbide into the Al-based matrix allowed the improvement in the basic mechanical properties of composites, wear and abrasion resistance, crack resistance, and many other characteristics.

Nanoparticulates are used increasingly in MMC production because they provide better properties of composite materials. Mechanical, tribological, and microstructural properties of the aluminum alloy reinforced by Al_2_O_3_ nanoparticulates with different weight contents were tested by Raturi et al. [9]. Properties tested were better compared to composites with the addition of microparticulates. It was described that when the content of nano-Al_2_O_3_ increased, they became more agglomerated and the residual porosity increased. The size of nanoparticulates in a metal matrix affects the MMC properties significantly, as it was revealed by Hassanzadeh-Aghdam et al. [10]. More dispersed particles lead to the improvement in these properties. Therefore, the conglomeration of nanoparticles is extremely undesirable, although it has been observed in a number of cases.

Composites based on iron and its alloys are much less studied. This is primarily due to the areas of such composites used—such as antifriction materials or materials with special properties. Composites based on an iron–chromium alloy with additions of Al_2_O_3_ particulates were considered by Saidatulakmar et al. [11]. Research results have shown that the best alumina content, when microhardness and wear resistance increase, is 20%. With its further increase, the phenomenon of agglomeration was observed, which worsened the interaction between the matrix and the additive.

Feldshtein and Dyachkova [12] described features of the structural, mechanical, and tribological properties of MMCs with ultrafine additives. Hard nano-additives in the base material increased its strength 1.5–3-fold. In comparison to the base material, coefficients of friction of MMCs were decreased 2–3 times, the seizure pressure was increased 2–5 times, and the wear rate was lower more than 2–4 times. Nanocomposites based on Cu-10Zn tombac with the addition of Al_2_O_3_ nanoparticulates were considered by Ramkumar et al. [13]. It was determined that the presence of nanoparticulates improved the sinterability, hardness, and compressive strength, as well as the tribological behavior, of the MMCs tested. A comparison of micro and nanocomposites of the same composition revealed a significant advantage of the latter. Zhang et al. described in [14] that iron matrix composite materials contain ferrite, pearlite, and WC, W_2_C, and Fe_3_W_3_C carbides. The wear resistance of composites was significantly higher compared to martensitic wear-resistant steel. The dominant wear of composites was abrasion and oxidation, in contrast to the micro-plowing mechanism characteristic of traditional steel.

The effectiveness of using two or more additives at the same time to improve the composite properties has been confirmed in many studies. The hybrid effect of CuO nanoparticulates and SiC microparticulates on the Aluminum Matrix Composites (AMC) microstructure and mechanical behavior was considered by Rajmohan et al. [15]. Tensile strength, microhardness, and density were improved with increasing content of nanoparticulates. The combined effect of Al_2_O_3f_ fibers and Al_2_O_3p_ particulates upon infiltration of the AlCu4 alloy was discussed by Wang et al. in [16], where a significant resistance to wear of the materials studied was shown. Studies of hybrid composites based on the AlSi alloy, obtained with the addition of carbon nano-tubes (CNTs) and SiC_p_, fulfilled by Carvalho et al. [17], showed that their combined effect on tensile strength, yield strength, and fatigue life was much higher compared to the effect of each additive separately. Composites based on the AZ31 magnesium alloy with Al_2_O_3_ and SiC additives were considered by Karthick et al. in [18]. Their density and microhardness increased compared to the starting material due to the silicon carbide presence. The alumina had a supporting role. Megahed et al. described in [19] the tribological features of the Al-based composite with hybrid reinforcing by alumina and silicon carbide particulates. This provided an improvement in hardness and wear rate, and the efficiency of SiC was higher compared to Al_2_O_3_. Mechanical properties and structures of MMCs on the basis of high-alloyed CrMnNi steel with MgO, ZrO_2_, and Al_2_TiO_5_ additives were described by Weigelt et al. in [20]. In the studied materials, higher strength and strain hardening were registered in comparison to base steel. With the addition of Al_2_TiO_5_, an interfacial interaction is observed, so the ZrO_2_ and Al_2_TiO_5_ mixture improved the general properties of MMCs.

Al-matrix composites with SiC and Al_2_O_3_ additives were studied by Ashwath et al. in [21]. The density, mechanical, and tribological properties of composites studied were improved. The best effect depended on the characteristics of the base material, but invariably for the 6 wt% Al_2_O_3_ additive. With a lower content of the additive, agglomeration of ceramic particles and a decrease in the density of the studied materials were observed. Changes in the properties of iron-based MMCs with WC, SiC, and graphite microadditives were studied by Feldshtein et al. in [22]. The hybrid effect of different additives on the composite properties was analyzed, and it was found that depending on the volume and composition of additives, changes in the base properties can be increased or decreased.

Summarizing the above, it can be claimed that the use of hybrid additives and nanoparticulates can significantly improve the properties of the MMCs. However, there is practically no information on the effectiveness of using hard ceramic nanoparticulates, and first, metal oxides and carbides, such as Al_2_O_3_ and SiC. In this paper, the effect of Al_2_O_3_, SiC, and C nanoadditives and their mixtures on the microstructure, strength, fracture features, and tribological behavior of iron-based sintered materials is described.

## 2. Materials and Methods

### 2.1. Powders, Nanoadditives, and Samples Preparation

**Figure 1:**
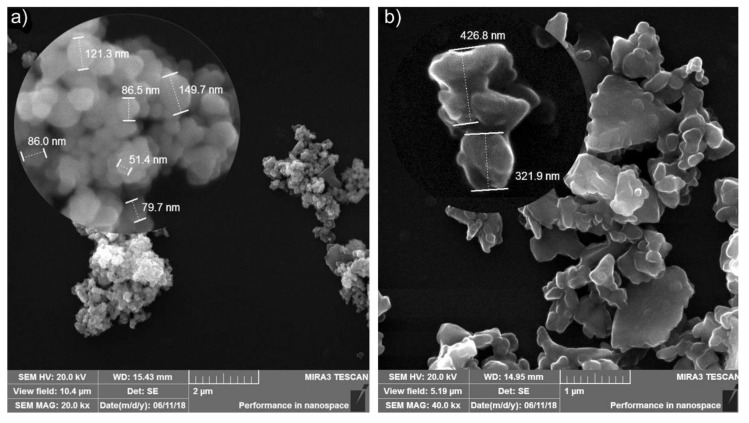
Al_2_O_3_ (**a**) and SiC (**b**) clusters.

The base material was prepared by using iron and carbon (graphite) powders. Alumina, silicon carbide particulates, and graphite particles were added to prepare the materials tested. The iron particles had an average size of less than 200 μm. The average size of graphite particles was 3 μm. Alumina particulates were of ~30–70 nm and silicon carbides had the average size of ~350 nm. Ceramic nanoparticulates were in the initial state-shaped clusters. As it was shown above [7,9,10,11,13], the presence of such clusters in the microstructure almost always leads to a deterioration in the basic characteristics of metal-matrix composites. Therefore, these clusters were crushed before producing samples. The features of ceramic clusters can be seen in Figure 1. A so-called “drunken barrel” mixer was used to prepare powder mixtures with a mixing time of 1.5 h. After that, the samples were molded with a hydraulic press under 500 MPa pressure. Then, they were sintered using a conveyor furnace in an endothermic gas atmosphere under temperatures of 900–1100 °C for 1 h. Under the forming conditions used, the residual porosity of 16–18% was ensured, both for the base material and for the MMCs investigated. When selecting MMC compositions, the microalloying principle was accepted as the base. In this case, a very small amount of additives is added to the base material, something like homeopathic drugs. The studies introduced in [12] revealed that the volume of additives should not exceed 1 wt%. The compositions tested are introduced in Table 1.

### 2.2. Materials and Samples Testing

A “Mira” scanning electron microscope (TESCAN ORSAY HOLDING, Brno-Kohoutovice, Czech Republic) was used to measure sizes of the ceramic particulates and base powders, as well as MMC fracture features. The JEOL JSM-5600LV scanning electron microscope (JEOL Ltd., Tokio, Japan) was applied to analyze wear behavior features. The SEM analysis was fulfilled with an INCA 350 analyzer (Oxford Instruments, Abingdon, UK). The X-ray diffraction method was used to analyze phase compositions and an ULTIMA IV X-ray diffractometer (Rigaku, Japan) was used. Tests were realized in CuKα radiation. Radiographies were registered at an angle range of 2θ = 3–150°. The XRD results were obtained using the “PDXL2” software (Rigaku, Japan). The analysis was performed for the material with the most complicated additive content: C + Al_2_O_3_ + SiC. The approach was adopted in that if compounds of certain chemical elements (Al or Si) are present in a multicomponent material (point 10 in Table 1), they will also be present in materials where they are present alone (points 2 and 3 in Table 1).

The Brinell tester was applied to measure the hardness. The strength properties were analyzed with a testing machine “Tinius Olsen H150K-U” (Tinius Olsen, Salfords, England) under a 2 mm/min rate of loading. The compression tests were realized in concordance with the ASTM C39/C39M-18 standard. Specimens had a cylindrical form of 10 mm diameter and of 16 mm height and the load acted on the end face. The flexural strength was tested in concordance with the ASTM C78/C78M-18 standard. Specimens had a parallelepiped form of 5 × 10 × 55 mm. This standard specifies that the bending test involves the plastic deformation of a specimen that is placed on two cylindrical supporting elements. The load is applied in the direction perpendicular to the reference plane at the midpoint of the specimen, and is hence named the “three-point flexural test.”

Tribological studies were conducted under concentrated contact conditions using the A-135 tester (UZ, Zielona Góra, Poland) that realizes the block-on roll friction scheme. Tests were carried out with a sliding speed of 0.45 m/s, load of 500 N, and test time of 40 min. Rollers (counter-bodies) were produced of C45 steel hardened to 45–51 HRC. The L-AN 68 machine oil (Orlen, Plock, Poland) was used as the lubricant with a flow rate of 30 droplets per minute. Momentary coefficients of friction and temperatures of the friction surface were measured using a computerized control system. Widths of wear areas were measured with a 0.001 mm accuracy using the Dino-Lite digital microscope (AnMo Electronics Corporation, Taipei, Taiwan), and values of the volume wear were calculated using equations introduced by Dyachkova et al. [23].

### 2.3. Design of Experiment Method

It is well known that the use of Design of Experiment (DOE) methods can minimize the cost of materials, time, finance, etc. In the case of research of multicomponent mixtures, it is especially important as it allows the consideration of not only the direct influence of each component but also their mutual influence on the researched characteristics of the new material. Such an approach becomes more and more feasible due to the use of modern software. In this paper, the experiments were carried out on the basis of one of the trends in the Design of experiments—experiments with mixtures according to Scheffe’s plane. In this case, strictly defined MMC compositions containing carbon (graphite), alumina, and silicon carbide nanoparticulates were investigated. The matrix of such a plan is presented in Table 1.

These plans are based on different mathematical models to describe mixture effects, and a cubic canonical polynomial for the third-order asymmetric curvature was used in this investigation as one of the options possible:

(1) $$\eta =\sum_{1\leq i\leq q}\beta_{i}x_{i}+\sum_{1\leq i<j\leq q}\beta_{ij}x_{i}x_{j}+\sum_{1\leq i<j\leq q}\gamma_{ij}x_{i}x_{j}(x_{i}-x_{j})+\sum_{1\leq i<j<k\leq q}\beta_{ijk}x_{i}x_{j}x_{k}$$

where β and γ—coefficients, *x_i_*, *x_j_*, *x_k_*—normalized volumes of C (*i*), Al_2_O_3_ (*j*), and SiC (*k*)—additives investigated, respectively.

The regression equation of this type allows the consideration of the maximum number of both single and complex effects of additives *i*, *j*, *k* on the parameter investigated η.

At each investigated point, tests were 3-fold reiterated to provide statistical reliability, and the Statistica 13 software (DOE option) was used for the mathematical processing of results. The standard errors calculated were of 5%. Response surfaces and isopleths were also generated using this software.

## 3. Results and Discussion

### 3.1. Composites Structures

**Figure 2:**
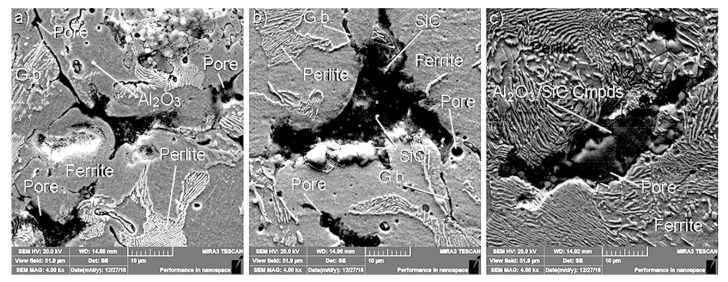
Microstructures of composites tested: (**a**) 1 wt% Al_2_O_3_ addition; (**b**) 1 wt% SiC addition; (**c**) 1/3 wt% Al_2_O_3_ + 1/3 wt% SiC + 1/3 wt% C addition; abbreviations used: G.b.—grain boundaries, Cmpds—complex compounds based on Al_2_O_3_ and SiC.

The analysis fulfilled using the SEM method revealed that nanoparticulates were located at the grain boundaries of the iron-graphite matrix. Their presence affects changes in the matrix microstructure (Figure 2). When either Al_2_O_3_ or SiC are present in the matrix singly, ferrite and perlite are the general microstructure components. However, when they are both present at the same time and in equal portions, perlite is predominantly present in the MMC microstructure.

Microstructure features affect mechanical and tribological properties of the MMCs under testing.

### 3.2. Substances Formed in MMCs

**Figure 3:**
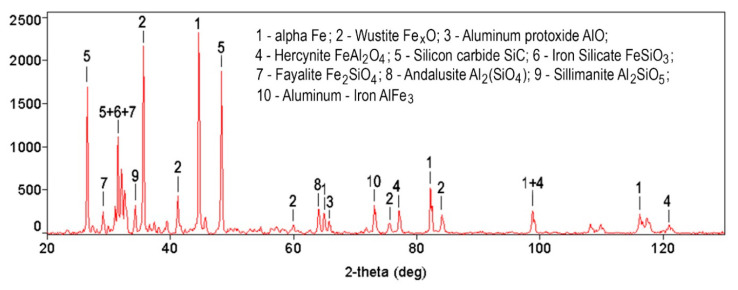
Chemical compounds formed in the FeGr1 + C + Al_2_O_3_ + SiC composite.

The alumina, silicon carbide, and graphite additives in the iron-based matrix lead to the shaping of some new compounds in the MMCs studied during the sintering process. Figure 3 introduces such compounds in the case of the material containing Al_2_O_3_ and SiC particulates. The effect of iron that had the greatest effect on the recorded diffraction patterns was neutralized due to the fact that the diffraction lines corresponding to ferrite and perlite were removed from the compounds list. However, iron-containing compounds have been taken into account.

### 3.3. MMCs Hardness

**Figure 4:**
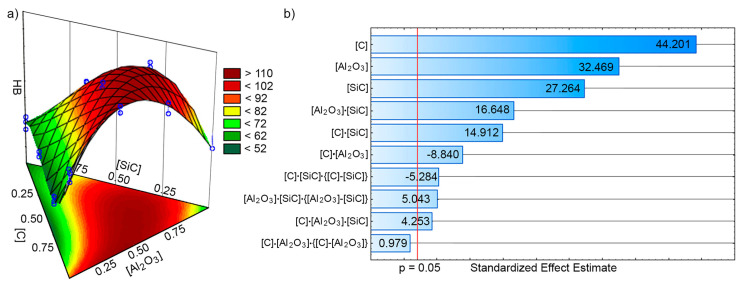
The effect of particulates content on the MMC hardness: (**a**) 3D HB function and isoplates; (**b**) Pareto chart.

The presence of mixture particulates in the Fe-based matrix influences its hardness considerably (Figure 4a). Hardness changes of the materials studied can be described by the model:

(2) $$\begin{array}{l} \mathrm{HB}=89.1667[C]+{65.5[\mathrm{Al}}_{2}O_{3}]+55[\mathrm{SiC}]+135.375[C]\cdot {[\mathrm{Al}}_{2}O_{3}]-80.25[C]\cdot \left[\mathrm{SiC}\right]+\\ {151.125[\mathrm{Al}}_{2}O_{3}]\cdot \left[\mathrm{SiC}\right]+279.75[C]\cdot {[\mathrm{Al}}_{2}O_{3}]\cdot \left[\mathrm{SiC}\right]-107.25[C]\cdot {[\mathrm{Al}}_{2}O_{3}]\cdot \left\{\left[C\right]-{[\mathrm{Al}}_{2}O_{3}]\right\}\\ +{102.373[\mathrm{Al}}_{2}O_{3}]\cdot \left[\mathrm{SiC}\right]\cdot \left\{{[\mathrm{Al}}_{2}O_{3}]-\left[\mathrm{SiC}\right]\right\} \end{array}$$

where [C], [Al_2_O_3_], and [SiC]—normalized values of the additives investigated, i.e., carbon, alumina, and silicon carbide, respectively.

It should be noted that the effect of particulates content on the MMC hardness is very complicated. This is evidenced by complex interactions between the contents of various components of the mixture. The statistical significance or insignificance of Equation (1) coefficients was revised using the Pareto chart (Figure 4b). The significance boundary is marked by a red line that corresponds to the chosen level of significance (0.05 in our case). The analysis of isoplates generated showed that the maximum hardness may be obtained in the case of the presence of ~0.75 wt% carbon, ~0.5 wt% alumina, and ~0.25 wt% silicon carbide.

### 3.4. MMCs Strenth

**Figure 5:**
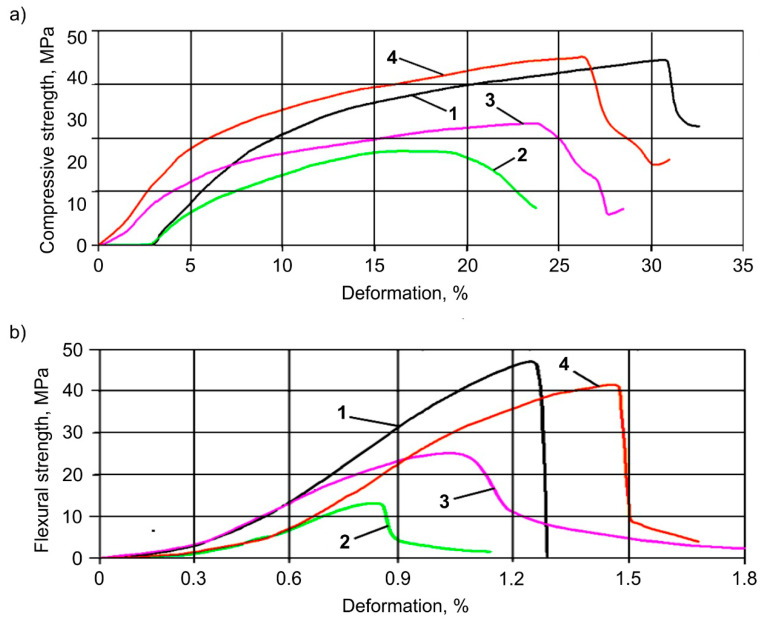
The stress–strain curves for MMCs containing C (1); Al_2_O_3_ (2); SiC (3); and C + Al_2_O_3_ + SiC mixture (4). (**a**) Compressive strength; (**b**) Flexural strength.

The influence of nanoadditives on stress–strain dependences, as well as bending and compressive stress limits of the MMCs under study, was analyzed, and the stress–strain curves are shown in Figure 5. In this figure, curve 1 relates to C addition, curve 2 relates to Al_2_O_3_ addition, curve 3 relates to SiC addition, and curve 4 to the C + Al_2_O_3_ + SiC mixture.

The introduction of alumina leads to the decrease in ultimate stresses, as well as the ultimate degree of deformation in the material tested. The introduction of silicon carbide has a similar effect, but to a lesser extent in comparison to the Al_2_O_3_ addition. The addition of carbon and the mixture of particulates has the best effect both on ultimate stresses and the ultimate degree of deformation.

**Figure 6:**
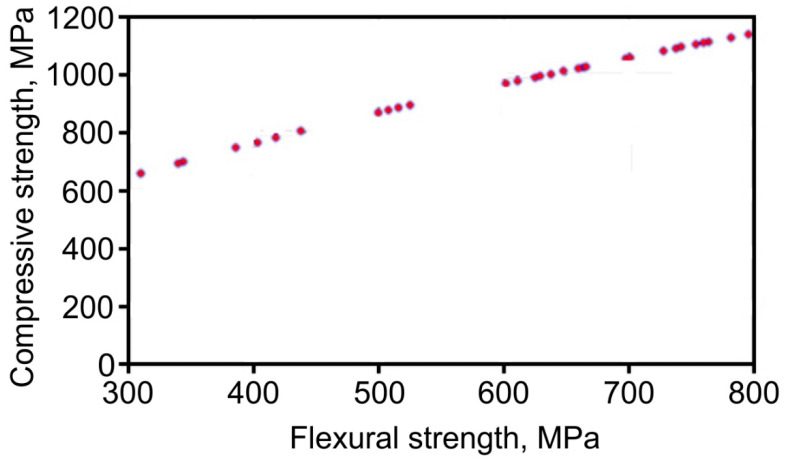
Relation between stresses studied.

Interconnection between the compression stress σ_c_ and flexural stress σ_f_ is introduced in Figure 6, and Equation (3) can be used to calculate the regression dependence:

(3) $$\sigma_{c}=23.043\sigma_{f}^{0.584}$$

Polynomial models to calculate the strength properties of the materials studied are introduced below:

(4) $$\begin{array}{l} \sigma_{f}=1041.08\left[C\right]+{856.32[\mathrm{Al}}_{2}O_{3}]+683.54[\mathrm{SiC}]+496.43[C]\cdot {[\mathrm{Al}}_{2}O_{3}]+\\ {1308.90[\mathrm{Al}}_{2}O_{3}]\cdot [\mathrm{SiC}]+1341.96[C]\cdot {[\mathrm{Al}}_{2}O_{3}]\cdot \left[\mathrm{SiC}\right]-1105.16\left[C\right]\cdot {[\mathrm{Al}}_{2}O_{3}]\cdot \left\{\left[C\right]-{[\mathrm{Al}}_{2}O_{3}]\right\} \end{array}$$

(5) $$\begin{array}{l} \sigma_{c}=663.00\left[C\right]+487.67{[\mathrm{Al}}_{2}O_{3}]+331.33\left[\mathrm{SiC}\right]+528.75\left[C\right]\cdot {[\mathrm{Al}}_{2}O_{3}]+\\ {1323.75[\mathrm{Al}}_{2}O_{3}]\cdot \left[\mathrm{SiC}\right]+1557.00[C]\cdot {[\mathrm{Al}}_{2}O_{3}]\cdot \left[\mathrm{SiC}\right]-1211.25\left[C\right]\cdot {[\mathrm{Al}}_{2}O_{3}]\cdot \left\{\left[C\right]-{[\mathrm{Al}}_{2}O_{3}]\right\} \end{array}$$

In Equations (4) and (5), the designations [C], [Al_2_O_3_], and [SiC] match up with normalized values of the additives studied, i.e., C, Al_2_O_3_, and SiC, correspondingly.

**Figure 7:**
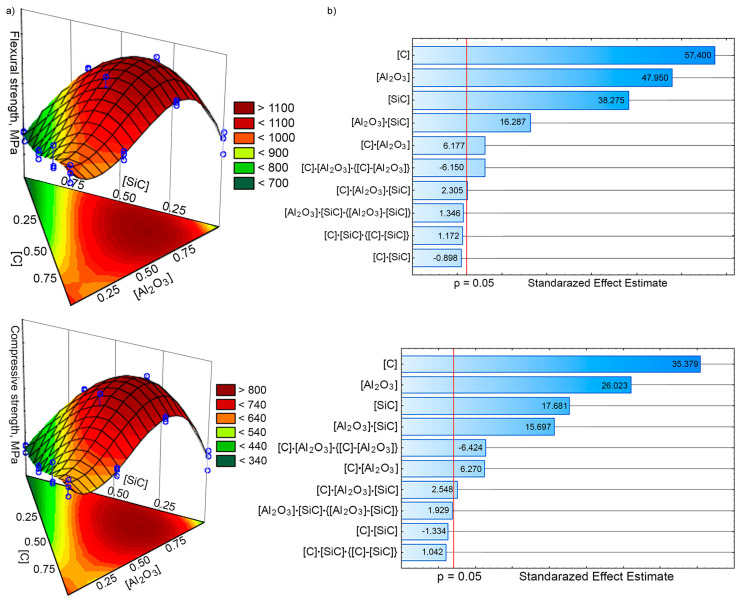
The influence of nanoadditives content on the strength of MMCs tested: (**a**) 3D functions and isoplates; (**b**) Pareto charts.

The effect of particulates content on the MMC strength is complicated. This is evidenced by complex interactions between the contents of various components of the mixture. The statistical significance or insignificance of Equations (4) and (5) coefficients was revised using the Pareto chart (Figure 7b). The analysis of isoplates generated showed that the maximum flexural strength and compressive strength may be achieved with the content of ~0.75 wt% carbon, ~0.75 wt% alumina, and ~0.35 wt% silicon carbide.

### 3.5. Fractures of Materials Studied

The interdependence between mechanical properties of MMCs and their compositions is complex because nanoadditives interact with the metal matrix at the micrometric and submicrometric levels. MMC fractures were analyzed based on Zehnder [24] and Kuna [25] investigations.

**Figure 8:**
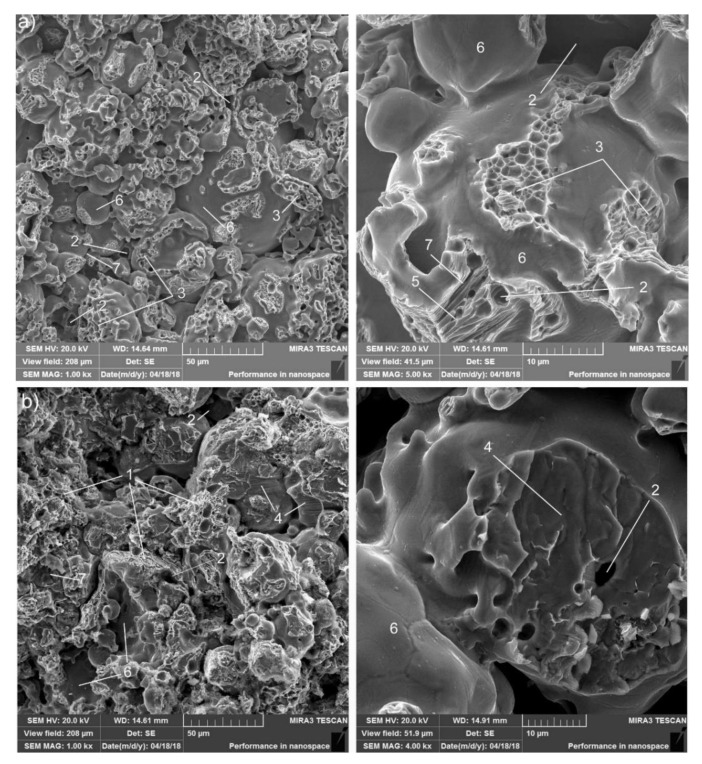
Fractographies of FeGr1 (**a**) and FeGr2 (**b**) material: 1—areas of quasi-brittle fracture; 2—pores; 3—areas of viscous fracture; 4—areas of brittle cleavage; 5—perlite grain destruction; 6—areas of inter-granular fracture; 7—microcrack.

The fracture of the FeGr1 base material is introduced in Figure 8a. Its structure consists of perlite and ferrite components. In the ferrite areas, zones of trans-crystalline viscous fractures are observed, whereas zones of quasi-brittle fractures are found in the perlite areas. Inter-crystalline fracture areas are observed in the boundaries of particulates, and pores in the base matrix are also observed. In some places, microcracks can be found.

The examination of FeGr2 material fracture identified trans-crystalline quasi-brittle fracture and also fragile faceted cleavage with facets of various sizes (Figure 8b). In this case, increased carbon content leads to the cementite formation in the base FeGr1 matrix. Areas of trans-crystalline quasi-brittle fracture and inter-granular fracture areas along the pores are also observed. Microcracks are present in the FeGr2 fracture too.

**Figure 9:**
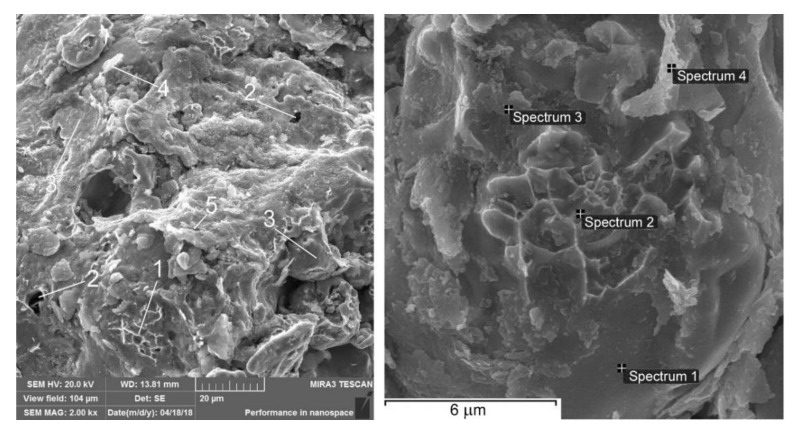
Fractographies of FeGr1 with 1 wt% Al_2_O_3_ particulates: 1—areas of transcrystalline quasi-brittle fracture; 2—pores; 3—areas of brittle cleavage; 4—Al_2_O_3_ particulate; 5—microcrack.

After adding 1 wt% alumina to the base material, the inter-granular fracture predominates (Figure 9). This is due to the fact that Al_2_O_3_ particulates are predominantly placed along the grain boundaries as described above. Besides that, trans-crystalline quasi-brittle and viscous fractures in the ferrite can be also noted. The SEM analysis of typical fracture submicroareas showed that alumina colonies are present on the surface of the fracture (spectra 4; 5 and Table 2). A conclusion may be drawn that Al_2_O_3_ nanoparticulates form agglomerates when MMC components are mixed before producing, regardless of the defragging. All this facilitates the reduction in strength of the material tested. In some places, microcracks can be found.

**Figure 10:**
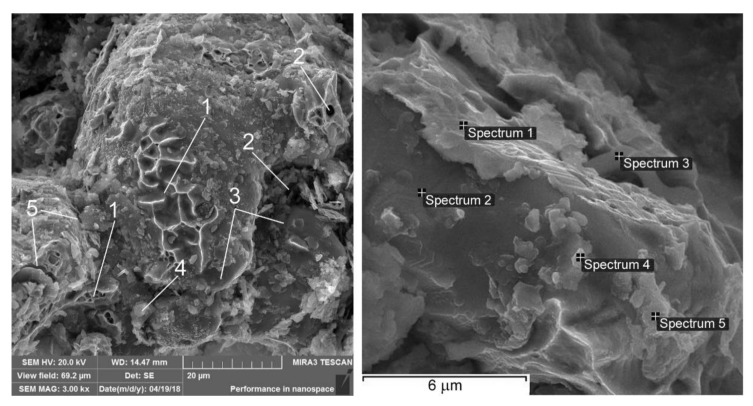
Fractographies of FeGr1 with 1 wt% SiC particulates: 1—trans-crystalline quasi-brittle fracture areas; 2—pores; 3—brittle cleavage areas; 4—SiC particulate; 5—microcracks.

After adding 1 wt% silicon carbide to the base material, the inter-granular fracture predominates (Figure 10). The reason for this is analogical to the case of the Al_2_O_3_ particulates presence in MMCs. Areas of inter-granular dimple destruction, probably in places where there are no carbides, are also observed. The SEM analysis of fracture submicroareas showed that silicon carbides attend on the surface of fracture in the form of single particulates and their agglomerates (Table 3). Microcracks can be observed in some places of the fracture.

**Figure 11:**
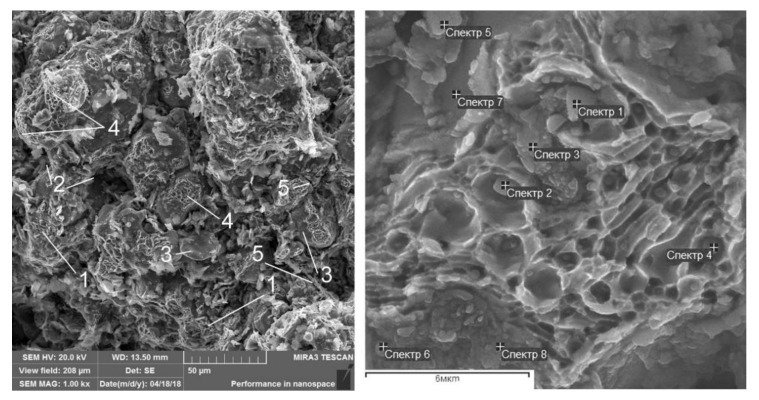
Fractographies of FeGr1 with the mixture of C, Al_2_O_3_, and SiC particulates: 1—areas of quasi-brittle fracture; 2—pores; 3—areas of inter-granular fracture; 4—areas of dimple viscous destruction; 5—microcracks.

The MMC is fine-granular when adding equal volumes of C, Al_2_O_3_, and SiC. Different types of fracture can be observed, particularly areas of quasi-brittle fractures, inter-granular fracture areas in the boundaries of particulates, and areas of dimple viscous destruction of the ferritic component, where there are no hard particulates (Figure 11). Dimples are shallow. Pores in the MMC matrix, as well as microcracks, can be found too. The SEM analysis results of fracture submicroareas for MMC after complex nanoparticulates addition are introduced in Table 4.

### 3.6. Tribological Behavior

**Figure 12:**
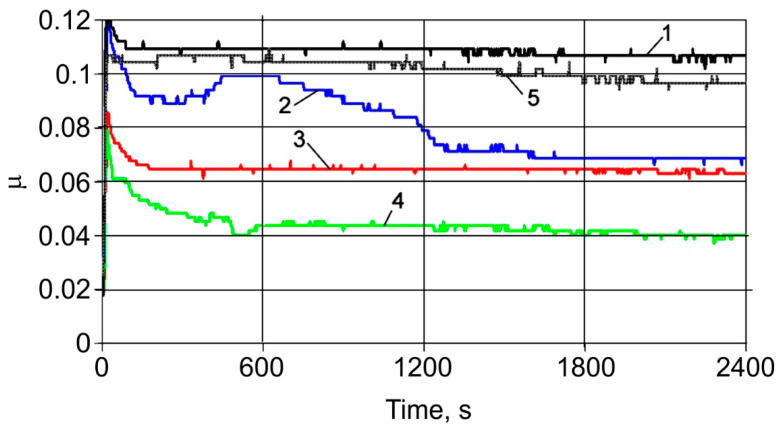
Changes in momentary coefficients of friction for MMC tested: 1—FeGr2; 2—1 wt% of Al_2_O_3_ additive, 3—1 wt% of SiC additive, 4—0.33 wt%C + 0.33 wt% Al_2_O_3_ + 0.33 wt% SiC additives, 5—FeGr1 base material.

Changes in the momentary coefficient of friction over time for typical MMC compositions are shown in Figure 12. In this figure, line 1 relates to the FeGr2 material, line 2 relates to 1 wt% of the Al_2_O_3_ additive, line 3 relates to 1 wt% of the SiC additive, line 4 relates to the mixture of C, Al_2_O_3_, and SiC nanoparticulates, and line 5 relates to the FeGr1 base material. It is easy to notice its rapid stabilization, which indicates a good running-in ability of composites with the addition of solid ceramic nanoparticulates. The stabilization time depends on the amount, composition, and features of the distribution of nanoparticulates added in the matrix of the MMCs. The most rapid stabilization of the coefficient of friction is observed with the introduction of SiC nanoparticles, and the minimum coefficient of friction is observed with the introduction of a complex additive of 0.33 wt% C + 0.33 wt% Al_2_O_3_ + 0.33 wt% SiC into the FeGr1 matrix. The presence of nanoadditives ensures the reduction in the momentary coefficient of friction by 1.5–2.5 times.

**Figure 13:**
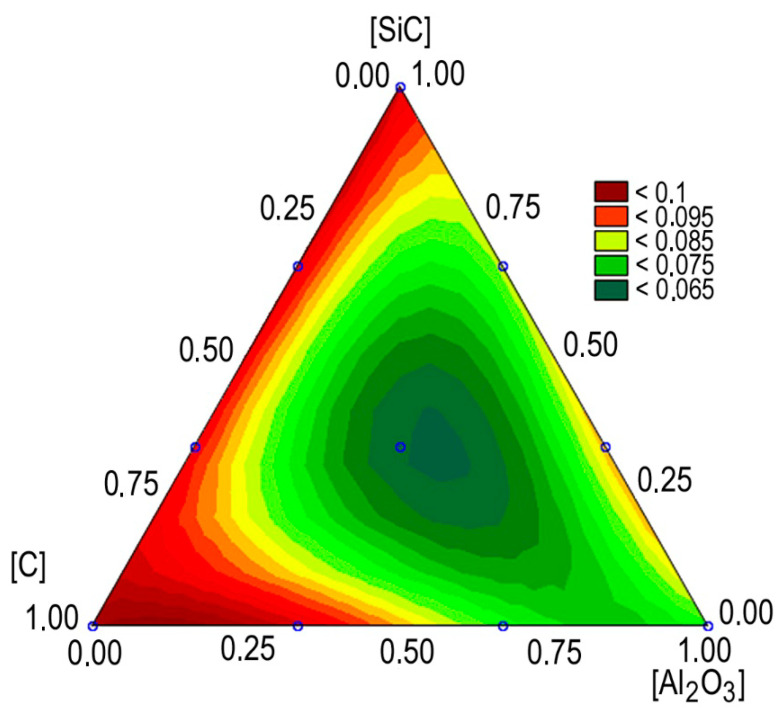
The effect of the MMC composition on the momentary coefficient of friction.

Figure 13 shows isopleths for the momentary coefficient of friction for MMCs tested by the method of simplex lattice design. It is easy to see that its minimum values are observed for a composition with approximately equal volumes of C, Al_2_O_3_, and SiC. An analysis of the Pareto chart showed that, in contrast to regression models for hardness and strength, the momentary coefficient of friction does not depend on the interaction of nanoadditives, but only their direct effect occurs.

**Figure 14:**
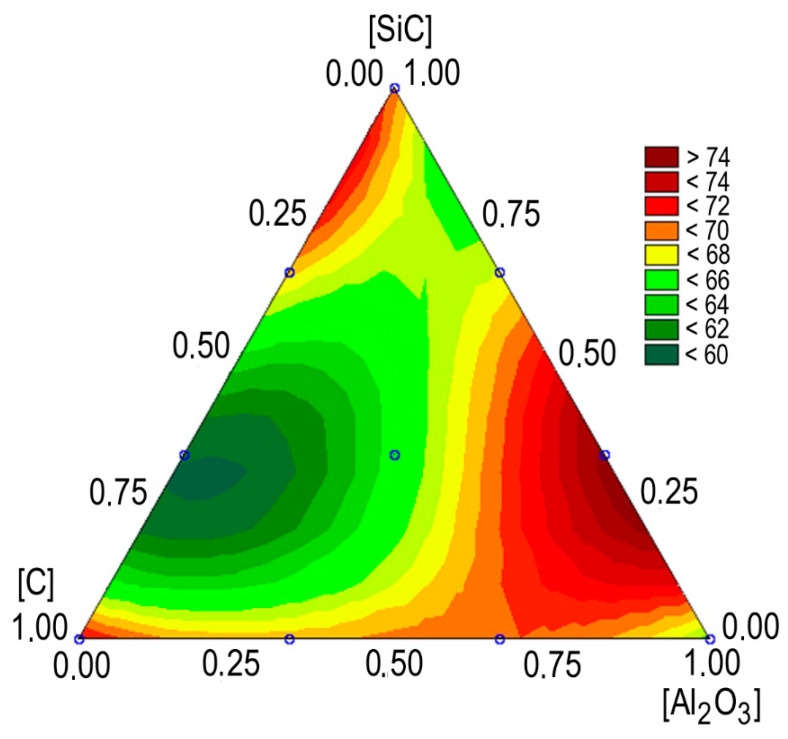
The effect of the MMC composition on the temperature of friction surface.

The effect of nanoadditives on the temperature in the friction zone is quite complex (Figure 14) and differs from similar dependencies for coefficients of friction, which is apparently due to the action of complex processes of forming new phases and structures on the surfaces of friction during the running-in and wear processes. Differences in temperatures reach 25%, the minimum temperature corresponds to the content of carbon addition in the composite, equal to 0.6–0.7 wt%, and silicon carbide adds to 0.25 wt%.

**Figure 15:**
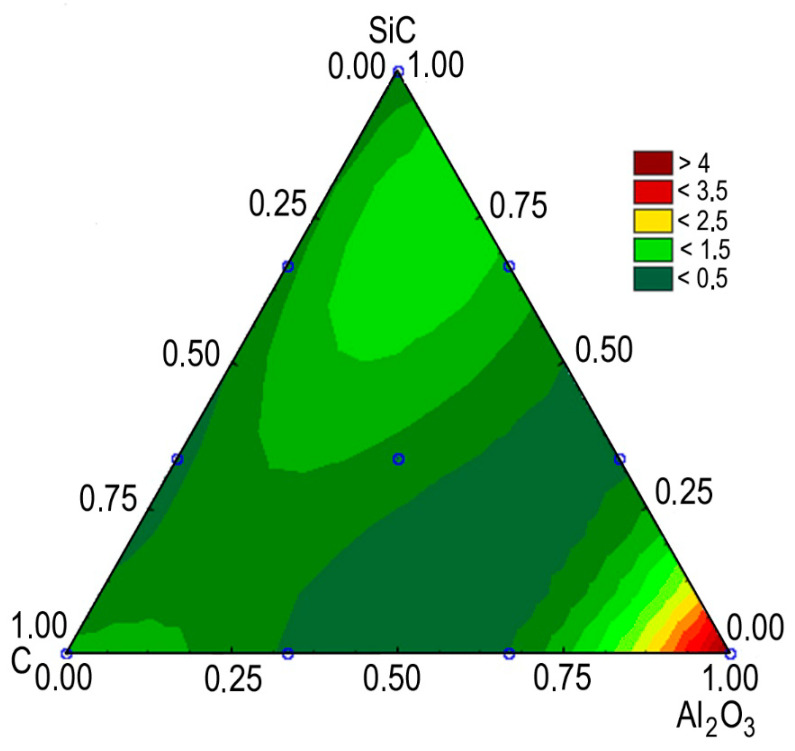
The effect of the MMC composition on the wear rate, mm^3^/h.

A diagram of the wear rate isopleths for the composites studied is shown in Figure 15. It is easy to see that the wear rate values substantially depend on the composition of the nanoadditives. The best results are achieved when 0.5–1 wt% C, 0.3–0.7 wt% Al_2_O_3_, and 0.3–0.5 wt% SiC are added to the base material. In the specified range of compositions, the wear rate was 4–5 times lower compared to the FeGr_1_ base material.

**Figure 16:**
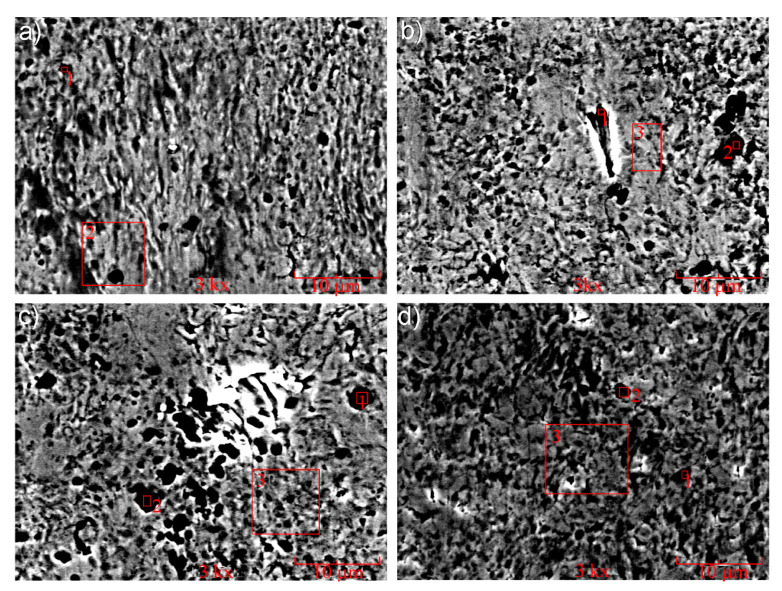
Worn surfaces of samples of MMCs tested: (**a**) 1 wt% of Al_2_O_3_ additive, (**b**) 1 wt% of SiC additive, (**c**) 0.33 wt% C + 0.33 wt% Al_2_O_3_ + 0.33 wt% SiC additives, (**d**) one of the compositions providing minimal wear rate (see Figure 15).

As it was noted above, the differences in the tribological characteristics of the composites studied are due to the peculiarities of the physical processes in the friction zone, which is indirectly confirmed by the SEM analysis of worn surfaces (Figure 16). Nanoadditives on the friction surface are identified both as individual nanoparticulates and as agglomerates (Table 5, Table 6, Table 7 and Table 8). The increased content of oxygen, apparently, is evidence of the increased content of machine oil on the friction surface, which accumulates in the micropores and lacunas formed during the friction process.

The study of the morphology of worn surfaces has shown that when the base FeGr1 material and base material with 1 wt% C addition are subjected to friction, typical types of wear can be observed—abrasion and adhesion processes. However, upon the introduction of Al_2_O_3_ and SiC nanoparticulates into the composite matrix, the nature of wear changes: A spongy-capillary texture, described in [26], is observed. The spongy-capillary effect occurs with the friction of composite porous materials under high loads and the presence of oil. In such conditions, pressures acting on the friction surfaces when running-in are high and go beyond the MMC strength. It provides high plastic deformations in the friction zone and the formation of different defects of the microstructure. Simultaneously, counter-body rotation affects impulse loads that exceed the fatigue limit and promote additional MMC hardening. This contributes to the movement of vacancies, which is presented in the complex MMC structure. The movement of micro- and nano-volumes of the material provides the formation of micropores and motion of dislocations. In this case, a block substructure and micro-capillaries are formed that enlarge the total volume of pores filled with oil. The oil may be injected cyclically into the capillary pores upon friction pair operation, which expands them and forms their favorable shape. These formations have a positive effect on the real contact of the composite with hardened steel. Such morphology of friction surfaces improves lubrication conditions and reduces the wear rate of friction surfaces, providing, in certain conditions, the effect of wearlessness.

## 4. Conclusions

In this research, the microstructures, strength, fractures, and tribological behavior of Fe-based composites with the addition of nanoparticulates were investigated. Powder metallurgy technologies, viz. pressing and sintering, were employed to prepare MMC samples. To determine the influence of the content of Al_2_O_3_, SiC, and C on the MMC properties, the simplex lattice design method was used. Conclusions can be made based on the results of research.

The addition of ceramic nanoparticulates into the base metal matrix considerably affects the MMC structures. These substances are located predominantly along the grain boundaries, but are also observed in the matrix grains. The XRD analysis reveals the appearance of a considerable quantity of new compounds. The presence of nanoparticulates has an impact on the hardness values, stress–strain relationships, and ultimate flexural and compression stresses. The maximum hardness may be obtained with adding ~0.75 wt% C, ~0.5 wt% Al_2_O_3_, and ~0.25 wt% SiC. The presence of nanoparticulates affects the flexural and compressive strength of MMCs. The maximum strength was attained when adding ~0.75 wt% C, ~0.75 wt% Al_2_O_3_, and ~0.35 wt% SiC.

Depending on the MMC composition, the conditions for its failure change. The introduction of Al_2_O_3_ and SiC nanoparticles mainly leads to inter-granular destruction due to the arrangement of particles along the boundaries of the grain; ceramic particulates can form conglomerates and be individually arranged. In the case of adding an equal volume of Al_2_O_3_, SiC, and C, the MMC is fine-granular. Pores in the MMC matrix, as well as microcracks, were observed in all fractures studied.

The presence of hard ceramic nanoparticulates contributes to the quick running-up ability of composites and ensures a decrease in the momentary coefficient of friction by 1.5–2.5 times. Its minimum values are observed for a composition with equal Al_2_O_3_, SiC, and C particulate contents. The wear rate of the composites studied significantly depends on the composition of the nanoadditives. The minimum values of wear rate, 4–5 times lower compared to the FeGr1 material, were registered for adding 0.5–1 wt% C, 0.3–0.7 wt% Al_2_O_3_, and 0.3–0.5 wt% SiC. The study of the morphology of worn surfaces has shown that with the introduction of Al_2_O_3_ and SiC nanoparticulates into the composite matrix, the specific spongy-capillary texture is observed there.

The presented research has shown the possibility of use of hybrid additives on the basis of hard ceramic nanoparticulates, as well as the simplex lattice design method for improvement of microstructural, mechanical, and tribological properties of MMCs. The approaches proposed in this paper will allow for more effective realization of studies in the area of MMCs and PMCs with different matrix materials.

## Figures and Tables

**Table 1:** The compositions of metal-matrix composites (MMCs) studied.

Composition Number	The Additives Composition (Compounds and Codes)		
	Gr (X1)	Al_2_O_3_ (X2)	SiC (X3)
0	1%	0%	0%
1	2%	0%	0%
2	1%	1%	0%
3	1%	0%	1%
4	1.33%	0.67%	0%
5	1.33%	0%	0.67%
6	1%	0.33%	0.67%
7	1.66%	0.33%	0%
8	1.66%	0%	0.33%
9	1%	0.67%	0.33%
10	1.33%	0.33%	0.33%

**Table 2:** Results of the SEM analysis of the fractural submicroarea of FeGr1 + 1 wt% of alumina.

Number of Spectrum	C, %	O, %	Al, %	Fe, %
1	1.1	5.6	1.2	The rest
2	1.3	5.0	1.2	The rest
3	1.3	25.2	15.4	The rest
4	2.1	21.8	14.0	The rest

**Table 3:** Results of the SEM analysis of the fractural submicroarea of FeGr1 + 1 wt% of silicon carbide.

Number of Spectrum	C, %	O, %	Si, %	Fe, %
1	42.7	0.6	6.0	The rest
2	43.8	0.4	3.3	The rest
3	39.5	1.3	0.4	The rest
4	41.6	1.6	1.9	The rest

**Table 4:** Results of the SEM analysis of the fractural submicroarea of the FeGr1+ mixture of C + Al_2_O_3_ + SiC.

Number of Spectrum	C, %	O, %	Al, %	Si, %	Fe, %
1	36.6	39.8	1.3	6.1	The rest
2	38.8	22.1	1.9	38	The rest
3	38.8	37.0	0.6	0.5	The rest
4	42.1	20.8	0.9	3.0	The rest
5	38.0	40.8	7.5	1.9	The rest
6	42.3	11.9	0.4	0.6	The rest
7	41.7	11.4	0.0	0.1	The rest
8	50.3	17.5	0.6	0.7	The rest

**Table 5:** The SEM analysis results of the FeGr1 + 1 wt% alumina worn surface (Figure 16a, areas 1 and 2).

Number of Area	C, %	O, %	Al, %	Fe, %
1	14.47	20.85	10.23	The rest
2	2.31	15.47	1.09	The rest

**Table 6:** The SEM analysis results of the FeGr1 + 1 wt% silicon carbide worn surface (Figure 16b, areas 1–3).

Number of Area	C, %	O, %	Si, %	Fe, %
1	22.01	7.13	0.47	The rest
2	37.99	3.79	34.63	The rest
3	2.71	6.39	0.11	The rest

**Table 7:** The SEM analysis results of the FeGr1 + C+Al_2_O_3_ + SiC mixture worn surface (Figure 16c, areas 1–3).

Number of Area	C, %	O, %	Al, %	Si, %	Fe, %
1	2.39	34.81	5.47	17.04	The rest
2	6.12	32.97	8.36	12.97	The rest
3	1.87	11.20	0.35	0.47	The rest

**Table 8:** The SEM analysis results of the worn surface of optimal composition MMC (Figure 16d, areas 1–3).

Number of Area	C, %	O, %	Al, %	Si, %	Fe, %
1	3.31	40.32	0.17	27.53	The rest
2	2.44	19.22	0.29	10.96	The rest
3	0.1	10.80	0.09	0.17	The rest
